# Auriculotherapy for reducing chronic spinal pain in health workers: a clinical trial*

**DOI:** 10.1590/1518-8345.6641.3954

**Published:** 2023-06-19

**Authors:** Bruna Xavier Morais, Oclaris Lopes Munhoz, Carlos Heitor Cunha Moreira, Leonice Fumiko Sato Kurebayashi, Luis Felipe Dias Lopes, Tânia Solange Bosi de Souza Magnago

**Affiliations:** 1 Universidade Federal de Santa Maria, Santa Maria, RS, Brasil.; 2 Becario de la Coordenação de Aperfeiçoamento de Pessoal de Nível Superior (CAPES), Brasil.; 3 Universidade Federal de Santa Maria, Departamento de Estomatologia, Santa Maria, RS, Brasil.; 4 Universidade de São Paulo, Escola de Enfermagem, São Paulo, SP, Brasil.; 5 Universidade Federal de Santa Maria, Departamento de Ciências Administrativas, Santa Maria, RS, Brasil.; 6 Universidade Federal de Santa Maria, Departamento de Enfermagem, Santa Maria, RS, Brasil.; 7 Becario del Conselho Nacional de Desenvolvimento Científico e Tecnológico (CNPq), Brasil.

**Keywords:** Musculoskeletal Pain, Chronic Pain, Auriculotherapy, Complementary Therapies, Clinical Trial, Quality of Life, Dolor Musculoesquelético, Dolor Crónico, Auriculoterapia, Terapias Complementarias, Ensayo Clínico, Calidad de Vida, Dor Musculoesquelética, Dor Crônica, Auriculoterapia, Terapias Complementares, Ensaio Clínico, Qualidade de Vida

## Abstract

**Objective:**

to evaluate the effectiveness of auriculotherapy in reducing chronic musculoskeletal pain in the spine of health workers.

**Method:**

a randomized, triple-blind clinical trial conducted with health workers diagnosed with chronic spinal pain. Eight sessions of auriculotherapy with seeds were applied, two *per* week. The outcomes were measured with the Numerical Pain Scale, Brief Pain Inventory, Rolland-Morris Disability Questionnaire and SF-36 instruments, in the 1^st^, 4^th^ and 8^th^ session, and in the 15-day follow-up period. Descriptive and inferential analyses were performed.

**Results:**

34 workers took part in the Intervention Group and 33 in the Control Group, and both presented reduced pain intensity (p>0.05). In the follow-up period, there was a greater reduction in the Intervention Group (3.32 ± 0.42), when compared to the Control Group (5.00 ± 0.43) (p=0.007). In quality of life, there was improved vitality (p=0.012) and limitation due to emotional aspects (p=0.025). The relationship between auriculotherapy, physical disability and pain interference did not differ between the groups (p>0.05). Medication use in the follow-up period remained unchanged in the Control Group (77.8%) when compared to the Intervention Group (22.2%) (p=0.013).

**Conclusion:**

auriculotherapy exerted the same effect between the groups on pain intensity, lasting longer in the follow-up period. There was an improvement in quality of life and a reduction in medication use. REBEC: RBR-3jvmdn.

## Abstract (es)

**Objetivo:**

evaluar la efectividad de la auriculoterapia para reducir el dolor musculoesquelético crónico de la columna vertebral de los trabajadores de la salud.

**Método:**

ensayo clínico aleatorizado, triple ciego, realizado con trabajadores de la salud diagnosticados con dolor crónico en la columna vertebral. Se aplicaron ocho sesiones de auriculoterapia con semillas, dos por semana. Los resultados fueron medidos con la Escala Numérica del Dolor, el Inventario Breve del Dolor, el Cuestionario de Discapacidad de Roland-Morris y los instrumentos SF-36, en la 1.ª, 4.ª, 8.ª sesión y *follow-up* a los 15 días. Análisis descriptivo e inferencial.

**Resultados:**

participaron 34 trabajadores del grupo intervención y 33 del grupo control, ambos mostraron una reducción en la intensidad del dolor (p>0,05). En el *follow-up* se registró una mayor reducción en el grupo intervención (3,32 ± 0,42) que en el grupo control (5,00 ± 0,43) (p=0,007). En cuanto a la calidad de vida, mejoró la vitalidad (p=0,012) y la limitación por aspectos emocionales (p=0,025). La relación entre auriculoterapia, discapacidad física e interferencia del dolor no difirió entre los grupos (p>0,05). El uso de medicamentos en el *follow-up* se mantuvo en el grupo control (77,8%) con respecto al grupo intervención (22,2%) (p=0,013).

**Conclusión:**

la auriculoterapia tuvo el mismo efecto en ambos grupos sobre la intensidad del dolor y duró más en el *follow-up*. Hubo una mejora en la calidad de vida y una reducción en el consumo de medicamentos. REBEC: RBR-3jvmdn.

## Abstract (pt)

**Objetivo:**

avaliar a eficácia da auriculoterapia na redução da dor musculoesquelética crônica na coluna vertebral de trabalhadores da área da saúde.

**Método:**

ensaio clínico randomizado, triplo cego, realizado com trabalhadores da saúde com diagnóstico de dor crônica na coluna vertebral. Aplicaram-se oito sessões de auriculoterapia com sementes, duas por semana. Desfechos mensurados com os instrumentos Escala Numérica da Dor, Inventário Breve de Dor, Questionário de Incapacidade de Rolland-Morris e SF-36, na 1ª, 4ª, 8ª sessão, e *follow-up* de 15 dias. Análise descritiva e inferencial.

**Resultados:**

participaram 34 trabalhadores no grupo intervenção e 33 no controle, ambos apresentaram redução da intensidade da dor (p>0,05). No *follow-up*, maior redução no grupo intervenção (3,32 ± 0,42), comparado ao controle (5,00 ± 0,43) (p=0,007). Na qualidade de vida, melhorou a vitalidade (p=0,012) e limitação por aspectos emocionais (p=0,025). Relação entre auriculoterapia, incapacidade física e interferência da dor não diferiu entre os grupos (p>0,05). O uso de medicamentos, no *follow-up*, manteve-se no grupo controle (77,8%) se comparado à intervenção (22,2%) (p=0,013).

**Conclusão:**

a auriculoterapia apresentou igual efeito entre os grupos na intensidade da dor, com maior durabilidade deste no *follow-up*. Houve melhora da qualidade de vida e redução do consumo de medicamentos. REBEC: RBR-3jvmdn.

**Highlights:**

**(1)** Auricular stimulation in true and false points (*sham*) exerts the same effect.

**(2)** There is greater durability of pain reduction in the follow-up period with the use of true points.

**(3)** Auriculotherapy enables the promotion of quality of life.

**(4)** Auriculotherapy reduced medication use in the workers under study.

## Introduction

Musculoskeletal Pain (MSP) is a consequence of repetitive stress injuries, traumas and mechanical overloads, among others^(1)^. Expressed by symptoms such as pain, tingling, paresthesia, sensation of heaviness, fatigue, numbness and limitation of movements^(2)^, it is classified into acute or chronic. As a rule, acute pain occurs in the initial phase of the disease, has a definite cause, is more localized and manifests itself for a short period of time; chronic pain has prolonged duration and clinical resolution, generally longer than three months^(3)^.

As a consequence of MSP chronicity, especially in the spine region, it can manifest affections that influence health and well-being. Among them, behavioral changes and physical functional disabilities stand out, which impose limitations in performing activities of daily living^(4)^ and/or in the work environment. In addition, there is a negative influence on physical and cognitive functions, quality of life and sleep, mood and changes in social behavior, including leisure and work activities^(5)^.

Among the workers affected by MSP, those from the health area working in the hospital environment stand out. In this context, there is exposure to several factors capable of predisposing to physical involvement or psychological distress^(6)^. The scientific literature shows high prevalence of MSP (from 53.8% to 83%) among health workers^(2,7-8)^.

Chronic MSP is related to the high number of absences from work^(2)^, resulting in work overload to other workers and increased costs for the institutions. From this perspective, research studies aimed at strategies to assist in reducing chronic MSP are important, such as complementary and integrative therapies^(3,9-10)^.

In this context, auriculotherapy stands out, an ancient practice based on the Traditional Chinese Medicine principles. It resorts to the stimulation of reflex points in the ear, using seeds, metallic spheres and/or crystals, semi-permanent and/or filiform needles^(11)^. From this starting point, it can generate a symptom-relieving action in organs or body regions, by fostering the release of endorphins into the bloodstream for pain relief^(3)^. Thus, it favors body homeostasis^(3,11)^, promoting psychological-organic regulation in the individual.

However, there is lack of studies evaluating auriculotherapy as an intervention to reduce MSP and improve quality of life in health workers. This was observed in an integrative review developed in 2019^(12)^ and updated in 2022. Of the 20 productions included in the evidence synthesis, an international study associated the reduction of chronic low back pain with the participants’ quality of life. No study covered all health professional categories.

In this perspective, we highlight the importance of developing experimental research studies aimed at promoting and recovering health, in addition to preventing diseases in health workers. This study will allow identifying the effect of auriculotherapy on the physical and psychological health of this population segment, related to MSP, physical disability and quality of life. This, the objective was to evaluate the efficacy of auriculotherapy in reducing chronic musculoskeletal pain in the spine of health workers.

## Method

### Study design

An experimental study with a randomized, triple-blind clinical trial design (patient, statistician and outcome evaluators), with a 1:1 allocation rate. The recommendations followed were those set forth in the Consolidated Standards of Reporting Trials (CONSORT)^(13)^ and registered in the Brazilian Registry of Clinical Trials (*Registro Brasileiro de Ensaios Clínicos*, REBEC), Code RBR-3jvmdn.

### Setting

A teaching hospital in the central region of the state of Rio Grande do Sul, Brazil.

### Period

The data were collected from March 2021 to January 2022.

### Population

Health workers, regardless of sector or professional category, active during the data collection period and who met the selection criteria.

### Selection criteria

Inclusion criteria: being available for the auriculotherapy sessions; medical diagnosis of chronic pain in the spinal region (cervicalgia, thoracic and lumbar pain), including the regions between the first cervical vertebra and the gluteal fold, lasting at least three months^(3)^; and minimum pain intensity of four on the Numerical Pain Scale. Exclusion criteria: being pregnant^(14)^; diagnosis of renal lithiasis with surgical indication^(15)^; reporting allergy to seeds; having inflammation in the atrium; and undergoing treatment for chronic MSP with alternative therapies.

### Definition of the sample

Sample calculation was performed by means of the unpaired sample formula, recommended for experimental studies^(16)^. The following were considered: n for the sample size; Sa and Sb for the standard deviation of the variable in each group^(17)^; Zα/2 for the α error value, usually 1.96 (5%); Zβ for the β error value, usually 0.84 (20%); and d, the minimum difference between the means^(17)^. The calculation resulted in 22 participants *per* group. An estimate of 30% was considered for possible losses, totaling a minimum of 29 participants *per*group.

### Study variables

Primary outcome: reduction in pain intensity. Secondary outcomes: physical disability and quality of life. Independent variables: sociodemographic characteristics, habits and health, and work-related data.

### Instruments used to collect the information

To characterize the workers (first phase of the study), sociodemographic data (gender, age), data on habits and health (tobacco consumption, medication use) and work-related data (weekly hour load, time in the profession and time working in the institution) were evaluated.

To evaluate the musculoskeletal symptoms, the Brazilian version of the Standardized Nordic Questionnaire^(18)^ was used in the first phase of the study. The Numerical Pain Scale^(19)^ and the Brief Pain Inventory^(20)^ were used to assess the pain intensity and interference to assess MSP, based on the application of auriculotherapy (second phase of the study). These instruments are used in research studies evaluating MSP^(2,8,12)^ and in clinical research^(3,21-22)^. For physical disability, the Brazilian version of the Roland-Morris Disability Questionnaire^(23)^ and the quality of life by the SF-36^(24)^ were employed.

### Data collection

In the first phase of the study, the workers answered the questionnaire on sociodemographic data, habits and health, work, the Brazilian version of the Standardized Nordic Questionnaire and the Numerical Pain Scale. Due to the COVID-19 pandemic, this questionnaire was first applied online (Google Forms)*.* After the second half of 2021, with the new distancing rules, printed questionnaires were made available.

The workers who met the selection criteria were invited to participate in the second phase of the study, corresponding to randomization and application of the auriculotherapy sessions. After agreeing to participate in the research, they were considered eligible and randomized into the Intervention Group (IG) or Control Group (CG).

Random allocation was performed in the IG or CG, using a computer program with numbers generated on the www.randomizer.org website. Block randomization (consisting of 6, 8, 10 and 12 randomized numbers) was carried out by the study coordinator. The numbers were given to the therapists in brown envelopes, numbered in sequence and sealed.

Eight auriculotherapy sessions were offered, two applications per week, and lasting a mean of 10 to 15 minutes. Auriculotherapy was developed with seeds, differing as follows: in the IG, the seeds were applied in the *shen men*, Kidney and Brainstem points and in points related to the outcome (cervical, dorsal or low back), these latter being stimulated in the front and back modality; in the CG, the seeds were applied in the Eye, Inner Ear, Seat and Urethra points. The auricular pavilion was alternated at each session in both groups.

To apply auriculotherapy, in both groups, the auricular pavilion was cleaned with cotton soaked in 70% alcohol, aiming to remove oiliness and promote disinfection of the auricular region. Mustard seeds were applied to the auricular pavilion, with the aid of a plastic sheet and rounded beige adhesive tape to fix them at the auricular points.

To locate the points related to the painful area in the IG (cervical, thoracic and/or low back), a manual locator was used for the reflex points proposed in the protocol. At the other IG and CG points, an auricular point locator (brand EL30 Finder Basic - NKL) was used, helping to provide greater precision for inserting the seeds.

The workers were instructed about the need for the seeds to remain in the points for three days and the care related to their daily stimulation, which should be done manually at least three times a day, 15 times, in each auricular point^(15)^. The sessions were scheduled in advance and offered in a room with privacy for physical examination of the ear, application of the therapy and answering the questionnaires.

Auriculotherapy was performed by three therapists, all with training and approximately five years of experience. As quality control, a data collection manual was developed with a description of the procedures to be followed. In addition to detailing the procedures, the therapists performed the first sessions together, in order to maintain the behaviors described in the protocol.

Four outcome evaluations were performed (in the 1^st^, 4^th^ and 8^th^ sessions, and after 15 days – Follow-up). The workers answered the Numerical Pain Scale, the Brief Pain Inventory, the Roland-Morris Disability Questionnaire and SF-36. These instruments were applied by a previously trained team, called “outcome evaluators” (who were blind regarding the groups to which the participants belonged).

### Data treatment and analysis

The data were entered into Excel^®^, with double checking. Subsequently, they were transferred to the PASW Statistic^®^ program (Predictive Analytics Software, from the Statistical Package for the Social Sciences – SPSS Inc., Chicago, United States – USA), version 18.0 for Windows. Descriptive and inferential statistics were used for data analysis. The descriptive statistics were performed through absolute (n) and relative (%) frequencies for the categorical variables, and the quantitative ones were analyzed through position and dispersion measures, according to data normality distribution or not, which was verified by means of the Shapiro-Wilk test.

The verification regarding homogeneity of the groups (sociodemographic data, habits and health, work and pain level) was based on the Chi-square or Fisher’s Exact tests for categorical variables and, for association between quantitative variables, the t test for independent samples or the Mann-Whitney test were performed.

The Numerical Pain Scale score varies from 0 (No pain) to 10 (Most severe pain) for the assessment of pain intensity, calculating the mean value. The Brief Pain Inventory has two final scores: Pain severity and Pain interference. The first one is evaluated through the mean of the “worst pain”, “weakest pain”, “pain mean value” and “pain intensity now” items. The second is evaluated by the mean of the “general activity”, “mood”, “ability to walk”, “work”, “relationship with other people”, “sleep” and “pleasure of living” items. The Roland-Morris Disability Questionnaire has 24 items, analyzed by the sum of affirmative answers. The score can vary from zero (No disability) to 24 (Severe disability). SF-36 has 36 questions and eight domains: Functional capacity, Physical aspects, Pain, Overall health status, Vitality, Social aspects, Emotional aspects and Mental health. The final score^(24)^ varies from zero (Worst general status) to 100 (Best general status).

The data resulting from the evaluation of the 1^st^, 4^th^ and 8^th^ session, as well as those from the follow-up period, were analyzed by intention-to-treat and protocol. Multiple imputation was used to define missing data over time. 20 imputations were performed to better converge with strong approximations of missing data^(25)^. Mixed linear models were used for analysis and comparison between the experimental groups. The best (unstructured) covariance structure was tested. The parameters of the model were estimated by maximum likelihood^(26)^. The statistical significance level for all tests was 0.05.

### Ethical aspects

The research was authorized by the institution and approved by its Research Ethics Committee, under registration number 3,897,861. The ethical precepts of research involving human beings were respected, according to Resolution No. 466/12. The participants signed the Free and Informed Consent Form in two copies and were informed about the study objectives and the possibility of withdrawing from the research.

## Results

**Figure 1 -:**
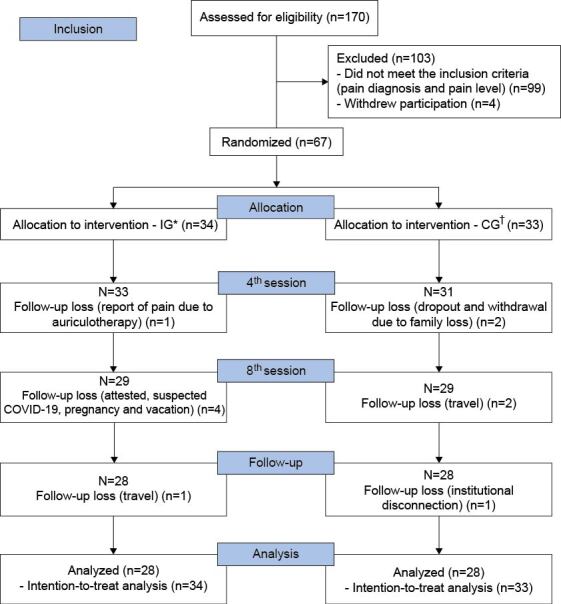
Flowchart corresponding to the study participants, adapted from CONSORT^(13)^. Santa Maria, RS, Brazil, 2021-2022

^*^Intervention Group; ^†^Control Group

A total of 67 professionals took part in the randomization phase. Of these, 34 were allocated to the IG and 33 to the CG (Figure1).

In the first phase of the study, there was high prevalence of MSP in the last year among those surveyed, with higher percentages of pain or discomfort in the neck (89.6%), shoulders (86.6%), lumbar spine (86.6%) and thoracic spine (68.2%). However, if pain or discomfort involved any disruption inside or outside the house, the highest percentages were in the lumbar spine (77.6%), neck (67.2%) and thoracic spine (57.6%) regions. Regarding the occurrence of this pain or discomfort in the last seven days, the workers presented them in the lumbar spine (82.1%), neck (68.7%) and shoulders (59.7%). In terms of pain intensity, there was a median of 7.00 (minimum of 4 and maximum of 10) and a mean of 6.85 (±1.44).

The comparison between the groups regarding the main characteristics evaluated pointed to homogeneity (p>0.05) (Table1).

**Table 1 -:** Characterization of the participants regarding sociodemographi, health and work-related data and pain characteristics in the intervention and control groups. Santa Maria, RS, Brazil, 2021-2022 (N=67)

Variables	Intervention Group	Control Group	p-value
**Gender N (%)**			
Female	30 (48.4)	32 (51.6)	0.356^*^
Male	4 (80)	1 (20)	
**Age Mean (±SD** ^†^ **)**	48.82 ± 9.22	48.42 ± 9.17	0.868^‡^
**Smoking habit N (%)**			
Ever smoked but stopped	8 (61.5)	5 (38.5)	0.371^*^
Never smoked	24 (51.1)	23 (48.9)	
Yes	2 (28.6)	5 (71.4)	
**Medication use N (%)**			
No	7 (53.8)	6 (42.2)	1.000^*^
Yes	27 (50)	27 (50)	
**Weekly hour load Mean (±SD** ^†^ **)**	35.59 ± 5.80	35.58 ± 5.24	0.920^‡^
**Time working in the profession Mean (±SD** ^†^ **)**	21.47 ± 9.47	21.03 ± 9.49	0.977^‡^
**Time working in the institution Mean (±SD** ^†^ **)**	14.15 ± 9.97	13.50 ± 9.20	0.975^‡^
**Pain intensity Median (Minimum-Maximum)**	7.00 (4-8)	7.00 (4-10)	0.806^§^

^*^Chi-Square test; ^†^Standard Deviation; ^‡^t test for independent samples; ^§^Mann-Whitney test

The impact of auriculotherapy on pain intensity, assessed by the Numerical Pain Scale (Table2), observed a reduction in pain intensity in both groups, until the evaluation at the 8^th^ session. There was a reduction in pain intensity in the 8^th^ session when compared to the first: 48% in the IG and 35% in the CG. However, no statistically significant reduction was observed between the groups (p=0.056). On the other hand, in the follow-up period there was a significant effect (p=0.007) of the intervention treatment, reducing pain intensity by 34% in relation to the control treatment.

In the pain intensity assessment, using the Brief Pain Inventory (Table2), it is observed that the IG presented a reduction in pain intensity after four auriculotherapy sessions and maintained it during the treatment and follow-up. On the other hand, the Cg presented a similar reduction in pain, more gradually and over the course of eight sessions, with no maintenance at the end (follow-up) (p=0.372).

**Table 2 -:** Mean (Standard Error) of intensity from the Numerical Pain Scale, pain intensity and interference according to the Brief Pain Inventory, and physical disability through the Roland-Morris Disability Questionnaire, according to the intervention and control groups. Santa Maria, RS, Brazil, 2021-2022

		Assessments	Intervention Group	Control Group
			Mean (SE^*^)	Mean (SE^*^)
Numerical Pain Scale	Pain intensity	1^st^ session (n=67)	5.94 (0.26)^Aa†^	5.88 (0.26)^Aa†^
		4^th^ session (n=64)	3.65 (0.41)^Ab†^	4.71 (0.43)^Ab†^
		8^th^ session (n=58)	3.11 (0.43)^Ab†^	3.80 (0.44)^Ac†^
		Follow-up (n=56)	3.32 (0.42)^Ab†^	5.00 (0.43)^Bab†^
Brief Pain Inventory	Pain intensity	1^st^ session (n=67)	5.57 (0.24)^Aa†^	5.45 (0.25)^Aa†^
		4^th^ session (n=64)	3.82 (0.36)^Ab†^	4.51 (0.37)^Ab†^
		8^th^ session (n=58)	3.43 (0.38)^Ab†^	3.70 (0.38)^Ac†^
		Follow-up (n=56)	3.66 (0.39)^Ab†^	4.18 (0.38)^Ac†^
	Pain interference	1^st^ session (n=67)	4.89 (0.36)^Aa†^	5.05 (0.37)^Aa†^
		4^th^ session (n=64)	2.84 (0.40)^Ab†^	3.24 (0.41)^Ab†^
		8^th^ session (n=58)	2.39 (0.40)^Ab†^	2.31 (0.40)^Ac†^
		Follow-up (n=56)	2.19 (0.40)^Ab†^	2.88 (0.39)^Ac†^
Roland-Morris Disability Questionnaire	Physical disability	1^st^ session (n=67)	12.12 (0.86)^Aa†^	11.73 (0.87)^Aa†^
		4^th^ session (n=64)	9.00 (1.00)^Ab†^	9.14 (1.02)^Abc†^
		8^th^ session (n=58)	7.47 (1.07)^Ab†^	7.77 (1.08)^Ac†^
		Follow-up (n=56)	8.18 (1.02)^Ab†^	9.06 (1.03)^Ab†^

Note: Mean values followed by the same capital letter (on the line) do not differ (p>0.05) considering the means between both groups in the same assessment condition. Mean values followed by the same lowercase letter (in the column) do not differ (p>0.05) considering the means within each group

^*^Standard Error; ^†^Linear mixed-model analysis (analysis by protocol)

Regarding pain interference in the activities of daily living (Table2), there was a reduction in the mean values in both groups (p>0.05). In the IG, this reduction takes place in all the assessments whereas in the CG, an increase in the follow-up mean is observed when compared to the evaluation at the 8^th^ session (mean difference of 0.58). A higher mean reduction between the 1^st^ session and the follow-up period was verified in the IG (55%) when compared to the CG (43%). The groups were similar in reducing physical disability during the 1^st^ to the 8^th^ session (p>0.05). In the follow-up period, the CG (9.06 ± 1.03) presented an increase in the mean when compared to the IG (8.18 ± 1.02).

Table3 presents the mean (Standard Error) corresponding to quality of life, according to SF-36.

**Table 3 -:** Mean (Standard Error) corresponding to quality of life, as *per* SF 36, according to the intervention and control groups. Santa Maria, RS, Brazil, 2021-2022

	Assessments	Intervention Group	Control Group
		Mean (SE^*^)	Mean (SE^*^)
Functional capacity	1^st^ session (n=67)	50.06 (3.68)^Ab†^	50.30 (3.70)^Ab†^
	4^th^ session (n=64)	54.40 (3.83)^Aab†^	55.14 (3.93)^Aab†^
	8^th^ session (n=58)	60.12 (4.01)^Aa†^	58.81 (4.04)^Aa†^
	Follow-up (n=56)	61.38 (4.04)^Aa†^	59.06 (4.07)^Aa†^
Limitation due to physical aspects	1^st^ session (n=67)	39.72 (6.45)^Ac†^	33.33 (6.49)^Ac†^
	4^th^ session (n=64)	55.03 (5.53)^Ab†^	52.25 (5.68)^Ab†^
	8^th^ session (n=58)	62.35 (7.15)^Ab†^	57.68 (7.22)^Aab†^
	Follow-up (n=56)	75.13 (5.78)^Aa†^	66.94 (5.83)^Aa†^
Pain	1^st^ session (n=67)	40.36 (2.18)^Ab†^	35.12 (2.18)^Ab†^
	4^th^ session (n=64)	50.10 (2.39)^Aa†^	44.65 (2.49)^Aa†^
	8^th^ session (n=58)	52.99 (3.09)^Aa†^	49.77 (3.12)^Aa†^
	Follow-up (n=56)	52.39 (2.87)^Aa†^	46.97 (2.88)^Aa†^
Vitality	1^st^ session (n=67)	56.32 (2.66)^Ab†^	48.03 (2.70)^Bb†^
	4^th^ session (n=64)	63.00 (2.99)^Aa†^	49.78 (3.07)^Bb†^
	8^th^ session (n=58)	63.21 (3.26)^Aa†^	55.22 (3.30)^Aa†^
	Follow-up (n=56)	64.21 (3.25)^Aa†^	55.64 (3.27)^Aa†^
Social aspects	1^st^ session (n=67)	57.88 (3.67)^Ab†^	52.34 (3.75)^Ac†^
	4^th^ session (n=64)	69.23 (3.25)^Aa†^	60.04 (3.38)^Ab†^
	8^th^ session (n=58)	70.64 (3.66)^Aa†^	69.43 (3.72)^Aa†^
	Follow-up (n=56)	75.31 (4.00)^Aa†^	66.93 (4.00)^Aab†^
Limitation due to emotional aspects	1^st^ session (n=67)	47.73 (6.99)^Ab†^	57.58 (7.01)^Aa†^
	4^th^ session (n=64)	78.71 (5.63)^Aa†^	60.13 (5.80)^Ba†^
	8^th^ session (n=58)	72.85 (7.11)^Aa†^	69.76 (7.15)^Aa†^
	Follow-up (n=56)	75.02 (6.41)^Aa†^	69.97 (6.45)^Aa†^
Mental health	1^st^ session (n=67)	66.71 (3.06)^Ab†^	58.18 (3.11)^Ab†^
	4^th^ session (n=64)	70.26 (2.82)^Aab†^	64.54 (2.92)^Aa†^
	8^th^ session (n=58)	72.51 (3.11)^Aa†^	66.48 (3.15)^Aa†^
	Follow-up (n=56)	73.88 (3.28)^Aa†^	67.57 (3.29)^Aa†^

Note: Mean values followed by the same capital letter (on the line) do not differ (p>0.05) considering the means between both groups in the same assessment condition. Mean values followed by the same lowercase letter (in the column) do not differ (p>0.05) considering the means within each group

^*^Standard Error; ^†^Linear mixed-model analysis (analysis by protocol)

Regarding the SF-36 instrument (Table3), the “vitality” domain presented a significant difference (p=0.012) between the IG and the CG. Differences were observed between the groups in all the sessions: 1^st^ (p=0.032), 4^th^ (p=0.003), 8^th^ (p=0.028) and follow-up (p=0.036).

In relation to the assessments within the groups, in the IG, the “vitality” domain showed a statistical difference between the 1^st^ and 4^th^ sessions (p=0.004), and between the 8^th^session (p=0.010) and the follow-up period (p=0.010). In the CG, the 1^st^ session differed from the 8^th^ (p=0.008) and from the follow-up period (p=0.013); and the 4^th^ session differed from the 8^th^ (p=0.018) and the follow-up period (p=0.048).

The “limitation due to emotional aspects” domain presented a difference between the IG and the CG in the evaluation of the 4^th^ session (p=0.025). Within the groups, there was an increase in the mean from the 1^st^ session in the IG, which differed from the 4^th^ session (p<0.001), the 8^th^ session (p=0.003) and the follow-up period (p<0.001).

The results generated (mean and standard error) in the intention-to-treat analysis of the instruments exposed are similar to the protocol analyses presented.

Table4 displays the participants’ distribution regarding medication use.

**Table 4 -:** Distribution of the participants on medication use (follow-up evaluation). Santa Maria, RS, Brazil, 2021-2022 (N=55)

Medication use	Intervention Group	Control Group
	N (%)	N (%)
Reduced the dosage	10 (71.4)^A*^	4 (28.6)^A*^
Maintained the dosage	4 (22.2)^A*^	14 (77.8)^B*^
Increased the dosage	-	1 (100)
No medication use	14 (63.6)^A*^	8 (36.4)^A*^

Note: Means followed by the same capital letter (on the line) do not differ (p>0.05) considering the frequencies between both groups in the same assessment condition

^*^Chi-square Test with *post hoc* (z Test-Bonferroni Method)

## Discussion

Auriculotherapy with seeds presented positive effects as a therapeutic practice in reducing pain intensity among health workers who had chronic pain in the spine. However, there was no statistical difference in the intergroup analysis. In other words, both groups obtained similar results during the treatment. In the follow-up segment, however, a significant 34% reduction was observed in the IG (3.32 ± 0.42) in relation to the CG (5.00 ± 0.43) (p=0.007). The IG maintained the treatment effect after the end, and the CG did not maintain the reduction in pain intensity.

A study conducted with German soldiers verified a reduction in chronic pain intensity between the groups (p=0.001), with the CG corresponding to usual care and the IG related to the application of auriculotherapy^(27)^. In line with this, a meta-analysis reasserts the positive effects of auriculotherapy in reducing the intensity of chronic pain in the spine, evidencing benefits in several evaluation parameters, whether subjective or physiological, proving to be a promising therapeutic tool in the relief of chronic musculoskeletal discomforts^(9)^.

According to the Brief Pain Inventory, pain intensity did not differ between the groups. However, it is noticed that the most expressive results occurred in IG; in other words, between the first session and the follow-up period, auriculotherapy showed a reduction in the mean pain intensity percentages: IG (34.3%) and CG (23.3%). The same result was obtained in relation to the evaluation of pain interference in activities of daily living, in which there was a reduction in the mean percentages in the IG (55%) and the CG (43%), although with no difference between them (p>0.05).

A similar result was found in a study from São Paulo conducted with individuals who had chronic pain in the spine. Auriculotherapy with semi-permanent needles in the treated (4.86 ± 2.79) and placebo (4.89 ± 2.74) groups presented a reduction in pain intensity (3.78 ± 3.49; 3.61 ± 3.49, respectively) and, in the CG (without intervention), there was no change in the mean values during the intervention period^(21)^. Regarding pain interference, it showed that auriculotherapy was effective in the treated group (Initial: 5.03 ± 2.54; Final: 1.59 ± 2.61) and placebo group (Initial: 4.92 ± 2.64; Final: 2 .82 ± 2.98), with better percentages for the treated group (mean reduction of 49.5%) when compared to placebo (mean reduction of 42.7%)^(21)^.

Given the above, it becomes necessary to consider that the health workers surveyed had high levels of chronic MSP in the spine (first phase of the study) and remained in an active work situation, that is, they continued to work even with high pain intensity. Some studies have deepened on the theme of presenteeism among health professionals, revealing permanence in activities even if they feel sick (either physically or emotionally)^(28-29)^.

The work context in which health workers are inserted requires knowledge, technical skills and a need for constant attention. They are exposed to biological and/or psychosocial risks, high workloads and direct contact with pain, suffering and losses^(30-32)^. These factors were potentiated in the pandemic context experienced since 2020, the period when this research was developed. Such situation favors the occurrence of symptoms such as anxiety, fear and uncertainty, which can negatively influence the perception of pain, and help trigger MSP episodes or intensify the symptoms^(33)^.

In addition to that, these professionals perform activities that expose them to ergonomic risks, such as the adoption of static postures, anterior inclination of the trunk and asymmetrical load lifting^(8)^, causing overload and muscle pain^(33)^. Associated with this, many workers adopt unhealthy life habits such as physical inactivity, sedentary lifestyle and immobility, responsible for causing tissue damage, such as atrophy and increased muscle stiffness, oftentimes related to low back and cervical pain^(33)^.

According to the Traditional Chinese Medicine assumptions, pain occurs due to *Qi*(energy) and *Xue* (blood) stagnation in the meridians. This stagnation can be due to internal pathogens, characterized by unbalanced emotions that affect the organs, and by external pathogens (pathogenic wind, cold and humidity) that, when invading the body and the individual is unable to eliminate them, lodge in the meridians, joints and muscles; in both cases, preventing free *Qi* and *Xue* flows^(10)^. Establishing a parallel with physiology, *Qi*circulates in the channels similarly to the venous and blood network. Thus, to promote their mobilization in the body, healthy lifestyle habits such as adequate nutrition and regular physical activity are important strategies. Therefore, these habits help strengthen the body both physically and psychologically, favoring vitality, willpower and longevity^(14)^.

Thus, when performing auriculotherapy, considering the viscero-somatic stimuli achieved by its application at the auricular points, the intention is to achieve regulation of this flow in the body, maintaining the *yin-yan* balance and the function of the internal organs^(13)^. Regarding neurophysiology, neurological reflexes occur by stimulating the auricular points which, as a result, release neurotransmitters and endogenous opioids (endorphins), responsible for regulating the pain control mechanisms^(34)^.

Simulation of the kidney auricular point, for example, has an important action for strengthening the low back region and the spine, and other analgesic and tranquilizing points help reestablish energy balance throughout the body^(35)^. Allied to this, the association of the *shen men* point has analgesic and anti-inflammatory actions and assists in the production and release of natural hormones, such as endorphin, directly linked to pain reduction and well-being in individuals^(34,36)^.

Regarding the physical disability assessment, it is considered that there is clinical improvement when the score is reduced by 30% from the baseline^(37)^. In this study, the workers who received the auriculotherapy protocol aimed at spinal pain showed a reduction in the mean values of 38.4% in the IG and of 33.7% in the CG between the first and eighth sessions. After evaluating the follow-up period, it was verified that the effect in the IG was long-lasting (32.5%) in relation to the CG (22.8%). Therefore, a clinical improvement is perceived, although without statistically significant difference between the groups.

A Brazilian study that applied auriculotherapy to Nursing professionals with chronic low back pain also evidenced no statistical difference. In relation to the physical disability scores between the IG (seeds), there was an Initial/Final mean difference of 1.4 ± 5.2 when compared to the CG (foam), which presented an Initial/Final mean difference of 1.8 ±2.4 (p<0.778)^(38)^.

The auriculotherapy practice can be developed through various materials and techniques. A systematic review evidenced that varied auriculotherapy modalities such as auricular acupressure, auriculotherapy with seeds and/or semi-permanent needles, with magnetic granules, can exert different effects even in the case of a similar condition, as the forms of stimulation of the auricular reflex points differ from each other^(9)^. An example of this is the use of semi-permanent seeds and needles. Whereas the former require manual stimulation of the participants (“dependent patient”), the latter maintains stimulation in a constant way by inserting the needle in the region.

In relation to the impact of the auriculotherapy protocol for chronic pain on quality of life, in this research, better results were observed in the physical and mental aspects in the IG when compared to the CG. However, only the “vitality” and “limitation due to emotional aspects” domains presented significant results. The “pain” domain did not display statistical differences between the groups, converging with the results of the other instruments that evaluated the musculoskeletal symptoms.

The “vitality” domain deals with the individual’s perception of their energy levels and fatigue for developing activities^(39)^, and the “limitation due to emotional aspects” domain refers to the extent to which emotional changes can interfere with their lives. Both domains presented an increase of 11.86% in the IG and of 3.64% in the CG; and pf 64.91% in the IG and 4.43% in the CG, respectively, in the 4^th^ session in relation to the 1^st^.

Chronic pain negatively influences quality of life in relation to mental health and maintenance of activities related to work and leisure^(40)^. The findings of this study show that auriculotherapy is a beneficial device to keep workers with positive vigor and energy levels. In addition to that, it proves to be advantageous practice for relieving depressive and/or anxious emotional symptoms^(11)^, avoiding interference in activities of daily living.

In this way, quality of life is promoted from the balance between a person’s physical, emotional and social aspects. A literature review evidenced that MSP severity exerts an influence on the physical and emotional dimensions, whereas pain chronicity interferes negatively in all the quality of life dimensions^(41)^. It is inferred that, from the relief of pain symptoms promoted by auriculotherapy, there is an improvement in some quality of life aspects.

A Brazilian study, which evaluated the effects of auriculotherapy (Control Group) and auriculotherapy associated with cupping therapy (Experimental Group) on the improvement of quality of life in adults with chronic low back pain, found significant changes in the social relations” and “psychological” domains, respectively^(40)^. In addition to that, it noticed an improvement of the “physical” domain in both groups^(40)^. It is inferred that auriculotherapy helps to improve quality of life, being developed individually and/or associated with other complementary therapies.

In relation to the consumption of medications for pain relief, it was observed that a higher percentage of the IG reduced their use and that the CG maintained it. Auriculotherapy reduced medication use in the workers who received it in the true points. These findings are important, as most of the participants suffered from musculoskeletal disorders, with the use of frequent medications.

Among the individuals with chronic pain, pharmacological treatments are the main method for controlling pain symptoms. However, their excessive use can trigger adverse effects and can cause harms in the gastrointestinal and nervous systems. Thus, these results reinforce the importance of offering new therapeutic practices, not pharmacological, that can contribute to relieving MSP^(4)^.

Based on the results presented and the improvement in the CG parameters evaluated in relation to the baseline, it is important to address the placebo effect. This effect can occur due to psychological mechanisms involved during the process, such as beliefs, previous experiences and personal motivation^(41)^. The bond between individual and therapist^(11)^ should also be considered, in view of the frequency of weekly meetings, which can increase positive expectations in relation to the intervention. In addition to that, through sensory and social stimuli, placebos can induce biochemical and cellular changes in people’s brains, being able to simulate and present beneficial responses^(41)^.

Similar placebo effects are observed in studies with pharmacological treatments. For example, a research study conducted with patients with migraines provided three envelopes, labeled as “Maxalt” (name of the medication used to treat migraines), “Placebo” and “Maxalt or Placebo”, each one containing one tablet of the active drug and another with placebo. The patients who used the placebo, called “Maxalt”, presented 30% effect on pain reduction; when they received the “Maxalt” labeled as “Placebo”, they showed a 38% improvement; and when they received the active medication labeled as “Maxalt”, they reported a 62% reduction^(42)^. Therefore, it is observed that the true intervention showed a better reduction; however, the effect provided by the placebo cannot be disregarded.

In relation to auriculotherapy, it is noted that there is a range of ear points and maps. In this way, finding *sham* (non-reactive) points is a difficult task. According to a French neurologist and physician^(43)^, *sham* points can produce positive effects due to ear innervations and the theory of embryological projection zones. For this author, the auricular pavilion is divided into three embryological membranes: ectoderm, mesoderm and endoderm. Each area has the potential to treat one type of pain. The “inner ear” and “eye” points would be on the ectodermal, an area that can reduce pain of a superficial nature. In addition, the “urethra” and “seat” points are located in the mesodermal projection zone, which is related to subcutaneous or muscular pain^(43)^.

In this research, in order to minimize this bias, the strategy was using a point locator that detects the internal resistance of the auricular pavilion. In the CG protocol, we searched for the region of the reflex point where there was less resistance, unlike the IG, where the one with more internal resistance was sought.

The pandemic context restricted the researchers entering the research locus, which represented a limitation. The possibility of failure in manual stimulation of the points under the participants’ responsibility can also be considered a limitation; this is because if the seeds are not pressed with the recommended frequency, there might be a reduction in the effect of auriculotherapy. In addition to that, it is considered that the moisture factor, due to the bath, can reduce local pressure and/or displace the seeds, which also reduces the effect.

As a contribution to science and the Nursing area, the objective was to highlight the benefits of a simple, fast and low-cost therapy with few side effects for health promotion to individuals, especially health workers, relieving high musculoskeletal pain levels. We understand that investing in promoting workers’ health, either through auriculotherapy, as in the case of this study, or with other Integrative and Complementary Practices (ICPs), will result in healthier workers, both physically and mentally.

## Conclusion

It was found that the auricular stimulus performed in true and false points (*sham*) exerted the same effect during all eight sessions of the study, more lasting in the follow-up segment for the IG, where a significant effect was shown in reducing pain intensity. Furthermore, auriculotherapy enabled the promotion of quality of life, especially in the “vitality” and “limitation due to emotional aspects” domains, as well as in medication use by the workers under study.
